# Prevalence, risk factors and health outcomes associated with polypharmacy among urban community-dwelling older adults in multi-ethnic Malaysia

**DOI:** 10.1371/journal.pone.0173466

**Published:** 2017-03-08

**Authors:** Li Min Lim, Megan McStea, Wen Wei Chung, Nuruljannah Nor Azmi, Siti Azdiah Abdul Aziz, Syireen Alwi, Adeeba Kamarulzaman, Shahrul Bahyah Kamaruzzaman, Siew Siang Chua, Reena Rajasuriar

**Affiliations:** 1 Department of Pharmacy, Faculty of Medicine, University of Malaya, Kuala Lumpur, Malaysia; 2 The Malaysian Elders Longitudinal Research (MELOR) Group, Faculty of Medicine, University of Malaya, Kuala Lumpur, Malaysia; 3 Centre of Excellence for Research in AIDS (CERiA), University of Malaya, Kuala Lumpur, Malaysia; 4 Pharmacy Department, University Malaya Medical Centre, Kuala Lumpur, Malaysia; 5 Faculty of Pharmacy, National University of Malaysia (UKM), Kuala Lumpur, Malaysia; 6 Department of Medicine, Faculty of Medicine, University of Malaya, Kuala Lumpur, Malaysia; 7 Peter Doherty Institute for Infection and Immunity, Melbourne University, Melbourne, Australia; University of Brescia, ITALY

## Abstract

**Background:**

Polypharmacy has been associated with increased morbidity and mortality in the older population.

**Objectives:**

The aim of this study was to determine the prevalence, risk factors and health outcomes associated with polypharmacy in a cohort of urban community-dwelling older adults receiving chronic medications in Malaysia.

**Methods:**

This was a baseline study in the Malaysian Elders Longitudinal Research cohort. The inclusion criteria were individuals aged ≥55years and taking at least one medication chronically (≥3 months). Participants were interviewed using a structured questionnaire during home visits where medications taken were reviewed. Health outcomes assessed were frequency of falls, functional disability, potential inappropriate medication use (PIMs), potential drug-drug interactions (PDDIs), healthcare utilisation and quality of life (QoL). Risk factors and health outcomes associated with polypharmacy (≥5 medications including dietary supplements) were determined using multivariate regression models.

**Results:**

A total of 1256 participants were included with a median (interquartile range) age of 69(63–74) years. The prevalence of polypharmacy was 45.9% while supplement users made up 56.9% of the cohort. The risk factors associated with increasing medication use were increasing age, Indian ethnicity, male, having a higher number of comorbidities specifically those diagnosed with cardiovascular, endocrine and gastrointestinal disorders, as well as supplement use. Health outcomes significantly associated with polypharmacy were PIMS, PDDIs and increased healthcare utilisation.

**Conclusion:**

A significant proportion of older adults on chronic medications were exposed to polypharmacy and use of dietary supplements contributed significantly to this. Medication reviews are warranted to reduce significant polypharmacy related issues in the older population.

## Introduction

The older population aged 60 years and above is expected to grow by 56% worldwide between 2015 and 2030, with the fastest growth in urban settings of developing regions [1]. In Malaysia, older populations have been projected to grow from 9.2% in year 2015 to almost a quarter of the total population (23.6%) by the year 2050 and this has been anticipated to add considerable demands on the countries’ health services [1, 2]. Among others, age related multi-morbidity and disability in older adults will increase the use of medications [3, 4] and subsequently the risk of polypharmacy which may be conceptually defined as the use of more medications than is medically necessary, though there is currently no universal definition for this in the literature [5]. Polypharmacy is a concern especially in older adults as they are prone to age- and disease-related pharmacokinetic and pharmacodynamic changes, and are thus more sensitive to drug therapy [6].

Polypharmacy in older individuals is strongly associated with multiple negative health outcomes which include increased healthcare costs [7], functional impairment, falls and fractures [8], harmful drug interactions [9] and adverse drug reactions [10, 11]. Intervention studies which focused on reducing polypharmacy among older persons either with the involvement of a pharmacist or a multidisciplinary team, have largely reported significant improvements in the quality of medication use [12–15]. Few studies have extended such observation to include health outcomes, but the findings have not been consistent [15, 16].

Despite a growing aging population and the known risks of increased medication related issues in this group, studies on polypharmacy among the older population in Malaysia have been limited to hospital settings and institutions of aged care [17–19]. Thus far, no studies have been found on older community dwelling individuals. In addition, only a few studies have included the use of dietary supplements in its assessment of polypharmacy despite their potential to cause clinically significant drug interactions and adverse drug events [9]. In multi-ethnic Malaysia, the use of herbal and dietary supplements are highly prevalent as reported in other Asian settings [20, 21] and these are often utilised as adjunct therapies for chronic health conditions [22–25]. Therefore, the present study aimed to determine the prevalence, risk factors and health outcomes associated with polypharmacy among urban community-dwelling older adults receiving chronic medications in Malaysia and further examined the role of dietary supplements in this context.

## Materials and methods

### Study design, setting and population

This was a baseline study conducted in the Malaysian Elders Longitudinal Research (MELoR) cohort. MELoR is a multi-disciplinary study conducted to explore the multi-dimensional aspects of aging faced by the urban community-dwelling older adults in Malaysia. Details of this cohort have been published previously [26]. Briefly, participants were individuals randomly identified through the Malaysian electoral roll at three representative urban parliamentary constituencies (Petaling Jaya South, Petaling Jaya North and Lembah Pantai), aged 55 years and above in 2013 and stratified by the three main ethnicities in Malaysia; Malays, Chinese and Indians. 8769 invitations were sent to the main ethnic groups and volunteers were invited through aged focused community groups. A total of 5815 participants with contactable addresses were identified through these efforts. Of these, only 3334 were eligible for the study (criteria below), had not moved to a new address or passed away. An age cut-off of 55 years was chosen because this age range enabled the analysis of issues in older individuals as they approached retirement. In Malaysia, the voluntary retirement age is 55 years and mandatory retirement age is 60 years. Individuals who were bed-bound, unable to be transported to the research centre for assessment and unable to communicate or answer questions due to advanced dementia or severe speech impediments, were excluded from the MELoR study. In addition, only individuals who were using at least one chronic medication, whether prescription or non-prescription and dietary supplements, were included in this study. Dietary supplements were defined as vitamins, minerals, amino acids, herbs and other botanicals as described previously [22].

Following written informed consent to participate in the study, all participants answered a questionnaire (either in English, Malay, Chinese or Tamil language) executed in the setting of their homes by trained interviewers. Subsequently, participants attended a study clinic in the University Malaya Medical Centre (UMMC) where further health assessments and biochemical screenings were performed. The questionnaire covered aspects of socio-demographic, general health, medical and medication history, healthcare utilisation and a series of validated questionnaires to assess falls, functional disability and quality of life (QoL). Recruitment for MELoR is ongoing and participants for the present study were selected among participants recruited consecutively from October 2013 till June 2015.

### Data collection

Participants’ baseline socio-demographic data and medical history were extracted from the MELoR database. Information on all medications used including dietary supplements (brand names and dosages) were verified by reviewing prescriptions and the actual medications during the home visits. Medications were classified according to the WHO Anatomical Therapeutic Chemical (ATC) classification system [27]. Dietary supplements which could not be classified according to the ATC system were coded as “others”. Two researchers independently classified the medications and any discrepancies were resolved by a third researcher, with the consensus of the initial two researchers.

### Study definitions and outcome measures

Polypharmacy was defined as the concurrent use of five or more medications [28]. These included prescribed, non-prescribed and over-the counter (OTC) medications including dietary supplements such as vitamins, minerals, herbals and other botanicals. Combined formulations (multiple active ingredients in a preparation) were considered as a single medication. This definition for polypharmacy is among the most widely used in the literature, is objective and allows comparability across different studies.

Potential inappropriate medications (PIMs) use was defined according to the American Geriatrics Society 2015 Updated Beers Criteria [29]. This guideline lists medications to be avoided in older adults in all settings except in hospice and palliative care, with the aim to reduce the risk of adverse drug events in older individuals. The updated Beers Criteria was used as it provides improved guidance on drug issues related to drug-drug interactions and medications to be avoided in older individuals with impaired renal function. Two researchers independently assessed participants’ medications for PIMs use and any discrepancies were resolved by a third researcher with their consensus. Assessment of kidney function were based on laboratory results obtained during the participants’ visit to UMMC for biochemical screening. As assessments for PIM use were done retrospectively, some assumptions were made when medical or medication history were incomplete for specific assessments. These assumptions are detailed in S1 File.

Potential drug-drug interactions (PDDIs) were assessed using the Thompson Micromedex® 2.0 interaction database (Truven Health Analytics Inc., Greenwood Village, CO, USA). Only interactions listed as major or contraindicated with clinically relevant and potentially serious consequences were included in the present study. PDDIs were assessed using this interaction database as it included potential interactions with dietary supplements which were not captured in the updated Beers Criteria [29].

Falls were defined based on self-reported responses to the question “Have you fallen in the past 12 months?”

Functional disability was assessed using the Modified Katz Index of Independence in Activities of Daily Living (ADL) [30] and Lawton Instrumental Activities of Daily Living (IADL) [31]. For ADL, participants were classified as dependent if they reported needing help with one or more daily living activities: walking, bathing, personal grooming, dressing, eating, getting from bed to chair and using the toilet. For IADL, participants who reported low function in one or more of the following activities were classified as dependent: using telephone, shopping, preparing food, doing housework, travelling, taking medicine and managing money.

Healthcare utilisation was assessed based on the participants’ reported number of visits to any of the following health facilities in the past 12 months: public hospital or clinic, private hospital or clinic and traditional or alternative medical health practitioners.

Quality of life (QoL) was assessed using 12 items which originated from the CASP-19 (Control, Autonomy, Self-realisation and Pleasure) questionnaire [32]. CASP-12 as previously described [33] is based on 12 Likert-like scale items which assessed four theoretically derived dimensions of QoL in older adults: control, autonomy, self-realisation and pleasure. Each item was numerically coded so that the most positive response was scored as 3 while the most negative response as 0. This outcome was analysed as a continuous variable (score range of 0–36), with lower scores associated with poorer QoL.

Chronic health problems were assessed based on self-reported diseases by participants and verified by the medications taken.

Payment method for healthcare services was assessed based on participants reported mode of payment when they utilized any healthcare facility in the preceding 12 months.

### Statistical analysis

Graphical tests were used to assess the normality of the data distribution. Parameters which were missing in 1% of participants or less were considered as negligible [34]. The number of medications taken as a count variable was used as a surrogate measure of polypharmacy in the analysis of risk factors and health outcomes associated with polypharmacy. Multivariate Poisson regression analysis was performed to identify risk factors associated with polypharmacy. Ordinal logistic regression was used to assess the health outcomes associated with polypharmacy specifically falls, functional disability, PIMs and PDDIs while the association of polypharmacy with healthcare utilisation and QoL were assessed using multivariate linear regression. In the analysis of risk factors associated with polypharmacy, only variables that produced a *p*<0.250 in the univariate analysis were included in the final multivariate model. For the analysis of health outcomes associated with polypharmacy, models were adjusted for the number of comorbidities, age, ethnicity and gender. All statistical analyses were performed using the Statistical Package for Social Science (SPSS) version 20.0 (SPSS Inc., Chicago, IL, USA) and Stata Statistical Software: Release 14 (StataCorp LP, College Station, TX). Statistical significance was defined as *p*<0.050.

### Ethics and sample size calculation

The study protocol was approved by the Medical Ethics Committee of University Malaya Medical Centre (MEC 943.6) and all participants provided informed written consent. Based on previous reported rates of polypharmacy in Malaysia in a hospital setting (46%), we estimated a minimum sample size of 400 would be required for this study using the binomial exact method with a 95% level of confidence and a 90% power.

## Results

A total of 1504 participants aged 55 years and above were recruited into the MELoR study from October 2013 through June 2015. 55% of eligible and contactable participants declined to participate either due to time constraints or inability to attend the research clinic. There was no difference in terms of gender and ethnicity (p>0.05 for all on chi-square analysis) among those who declined to participate compared to those included in the study. Of the 1504 recruited, 248 individuals did not receive any medications and hence, only 1256 participants were included in the present study.

### Characteristics of participants

The median (interquartile range, IQR) age of the participants was 69 (63–74) years, ranging from 55 to 97 years. The participants were almost evenly distributed among the three main ethnic groups in Malaysia that is, Indian (35.6%), Chinese (33.3%) and Malay (30.5%). The median (IQR) number of comorbidities were 3 (2–4) and the maximum reported was 11. A summary of the participants’ characteristics is shown in Table 1. The three most common chronic health conditions reported were cardiovascular disorders (1002 participants; 79.8%), endocrine disorders (472; 37.6%), bone and joint disorders (359; 28.6%). The three most commonly used medication classes were those that act on the cardiovascular system (1030; 82.0%), alimentary tract and metabolism (860; 68.5%), blood and blood forming organs (371; 29.5%). A complete list of medication classes used are detailed in S1 Table.

**Table 1:** Characteristics of all participants included in the study (n = 1256) as well as those with polypharmacy (n = 576) and dietary supplement users (n = 715).

**Characteristics**	**Total, n(column %) or median (IQR)**	**Polypharmacy, n(row %) or median (IQR)**	**Dietary supplement users, n(row %) or median (IQR)**
Total	1256 (100.0)	576 (45.9)	715 (56.9)
Age (n = 1256)	69 (63–74)	71 (66–76)	70 (64–75)
55–59	124 (9.9)	39 (31.5)	76 (61.3)
60–64	243 (19.4)	79 (32.5)	126 (51.9)
65–69	279 (22.2)	122(43.7)	160 (57.3)
70–74	300 (23.9)	151 (50.3)	178 (59.3)
75–79	202 (16.1)	118 (58.4)	119 (58.9)
80+	108 (8.6)	67 (62.0)	65 (60.2)
Gender (n = 1256)			
Male	532 (42.3)	259 (48.7)	269 (50.6)
Female	724 (57.7)	317 (43.8)	446 (61.6)
Ethnic (n = 1255)			
Malay	383 (30.5)	137 (35.8)	133 (34.7)
Chinese	418 (33.3)	170 (40.7)	294 (70.3)
Indian	447 (35.6)	265 (59.3)	284 (63.5)
Others	7 (0.6)	4 (50.0)	4 (50.0)
Highest education level (n = 1252)			
Primary or No Formal	326 (26.0)	133 (40.8)	121 (37.1)
Secondary	538 (43.0)	232 (43.1)	317 (58.9)
Tertiary	388 (31.0)	210 (54.1)	275 (70.9)
Employment status (n = 1255)			
No	1021 (81.4)	483 (47.3)	572 (56.0)
Yes	234 (18.6)	93 (39.7)	142 (60.7)
Payment method for healthcare service in the last 12 months^†^ (n = 1145)			
Used free service	56 (4.9)	32 (57.1)	34 (60.7)
Self/Out of pocket	591 (51.6)	252 (42.6)	332 (56.2)
Employer	292 (25.5)	156 (52.7)	156 (53.4)
Relatives	127 (11.1)	64 (50.4)	67 (52.8)
Welfare	112 (9.8)	62 (55.4)	67 (59.8)
Insurance	13 (1.1)	4 (30.8)	10 (76.9)
Non-governmental/Religious organizations	4 (0.4)	3 (75.0)	4 (100)
Chronic health problems^†^			
(n = 1256)			
Cardiovascular disorders	1002 (79.8)	607 (50.6)	519 (51.8)
Endocrine disorders	472 (37.6)	296 (62.7)	229 (48. 5)
Bone and joint disorders	359 (28.6)	211 (58.8)	246 (68.5)
Urologic disorders	321 (25.6)	170 (53.0)	182 (56.7)
Ophthalmic disorders	237 (18.9)	130 (54.9)	134 (56.5)
Respiratory disorders	104 (8.3)	58 (55.8)	53 (51.0)
Oncologic disorders	84 (6.7)	47 (56.0)	72 (85.7)
Gastrointestinal disorders	64 (5.1)	45 (70.3)	43 (67.2)
Renal disorders	39 (3.2)	30 (76.9)	21 (53.8)
Neurologic disorders	22 (1.8)	14 (63.6)	12 (54.5)
Falls in the last 12 months (n = 1256)	294 (23.4)	146 (49.7)	151 (51.4)
Functional disability			
Katz ADL	44 (3.5)	25 (56.8)	27 (61.4)
Lawton IADL	388 (30.9)	210 (54.1)	192 (49.5)
PIMs use (n = 1256)	400 (31.8)	271 (67.8)	177 (44.3)
PDDIs (n = 1256)	281 (22.4)	215 (76.5)	141 (50.2)
Smoking status (n = 1236)			
Never	985 (79.7)	446 (45.3)	584 (59.3)
Current smoker	96 (7.8)	36 (37.5)	40 (41.7)
Ex-smoker	155 (12.5)	87 (56.1)	78 (50.3)
Alcohol consumption (n = 1250)			
Never	889 (71.1)	388 (43.6)	467 (52.5)
Yes	280 (22.4)	147 (52.5)	204 (72.9)
Used to	81 (6.5)	37 (45.7)	40 (49.4)
More than 1 comorbidity	979 (78.0)	521 (53.2)	636 (65.0)
Using dietary supplements	715 (56.9)	401 (56.1)	-
Quality of life (QoL) (n = 124), CASP 12 score	28 (24‒31)	28 (24‒31)	28 (25–31)
Number of visits to any healthcare facility in the last 12 months^†^ (n = 1202)	4 (2‒6)	4 (2‒7)	3 (1–6)
Number of comorbidities (n = 1256)	3 (2‒4)	3 (2–5)	3 (1–4)
Number of prescribed and non-prescribed drugs (n = 1256)	3 (1‒5)	5 (3–6)	2 (1–4)
Number of dietary supplements (n = 1256)	1 (0‒2)	2 (0–3)	2 (1–3)
Total number of medications (n = 1256)	4 (2–6)	6 (5–8)	5 (3–7)

IQR: interquartile range; ADL: activities of daily living; IADL: instrumental activities of daily living; PIMs: potential inappropriate medications; PDDIs: potential drug-drug interactions

†More than one choice can be chosen.

### Prevalence of polypharmacy and supplement use

A total of 5804 medications (prescribed or non-prescribed medications and dietary supplements) were used, with a median of 4 (2–6) medications per person and a maximum of 20. The prevalence of polypharmacy was 45.9% with 576 individuals using at least five medications. Among those with polypharmacy exposure, 499 (86.6%) received 5 to 9 medications, 65 (11.3%) received 10 to 14 medications while 12 (2.0%) received 15 or more.

The percentage of participants using 5 medications or more increased with age (Table 1). Polypharmacy was also more prevalent among Indian participants compared to the Malay and Chinese. In terms of chronic health conditions, more than 70.0% of the participants who reported gastrointestinal, renal and psychiatric disorders were also taking 5 medications or more. In addition, 76.0% of the participants with PDDIs were classified in the polypharmacy cohort and also 67.8% of those with PIMs. A majority of the participants (54.4%) who reported having functional disability, assessed using either Katz ADL or Lawton IADL, and almost half of those who reported having falls, were categorised in the polypharmacy group.

More than half of the participants (56.9%) reported the use of at least one dietary supplement, with the median (IQR) of 1 (0–2) in the total cohort and 2 (0–3) in the polypharmacy group. Dietary supplements made up a total of 1782 or 30.7% of the total medications used (details in S2 Table) and were utilised by 401 participants (69.6%) who had polypharmacy exposure. When analysed by ethnic background, the proportion of Chinese who reported the use of dietary supplements was almost double that of the Malays (Table 1). The chronic conditions associated with the highest usage of dietary supplements were oncologic disorders, followed by bone and joint disorders, and gastrointestinal disorders. Among participants with PDDIs and PIMs, 50.2% and 44.3% respectively, reported the use of supplements.

### Risk factors associated with polypharmacy

Risk factors associated with increasing medication use as a surrogate of polypharmacy are summarised in Table 2. Multivariate Poisson regression analysis identified an increasing number of medications use as age increased compared to those aged 55‒64 (p<0.001) when adjusted for the other variables in the model. Malays (incidence rate ratio, IRR 0.89; 95% confidence interval, CI: 0.30, 0.94,) and Chinese (IRR 0.83; 95% CI: 0.78, 0.88) had a lower incidence rate ratio of medication use when compared to Indians (p<0.001) whilst all the other variables were constant. Similarly, when the other variables were held constant in the model, male participants had an incidence rate ratio (for the number of medications taken) of 1.09 times that of female participants (an increase of 9%). As participants increased the number of dietary supplements taken, they had a 19% increase in the total number of medications they were taking (IRR 1.19; 95% CI: 1.15, 1.23, *p*<0.001). Similarly, as the number of comorbidities increased, there was also an increased use of medications (IRR 1.14; 95% CI: 1.11, 1.17, *p*<0.001). Specifically, participants who had cardiovascular disorders (IRR 1.23; 95% CI: 1.14, 1.33, *p*<0.001), endocrine disorders (IRR 1.47; 95% CI: 1.31, 1.64, *p*<0.001) and gastrointestinal disorders (IRR 1.13; 95% CI: 1.03, 1.24, *p* = 0.010) had a higher incidence rate ratio for the number of medications taken compared to those without these disorders. However, those with urologic disorders were associated with a lower number of medication use.

**Table 2:** Risk factors associated with polypharmacy among urban community-dwelling older adults.

**Variables**	**Univariate Poisson regression**		**Multivariate Poisson regression**	
	Crude IRR (95%CI)	*p-value*	Adjusted IRR (95%CI)	*p-value*
Number of dietary supplements	1.13 (1.11–1.15)	**<0.001**	1.19 (1.15–1.23)	**<0.001**
Number of comorbidities	1.15 (1.13–1.16)	**<0.001**	1.14 (1.11‒1.17)	**<0.001**
Ethnic		**<0.001**		**<0.001**
Indian (reference)	1.00		1.00	
Malay	0.73 (0.67‒0.79)		0.89 (0.30‒0.94)	
Chinese	0.77 (0.71‒0.83)		0.83 (0.78‒0.88)	
Age		**<0.001**		**<0.001**
55‒59 (reference)	1.00		1.00	
60‒64	1.05 (0.93–1.21)		1.05 (0.95‒1.15)	
65‒69	1.27 (1.11‒1.44)		1.11 (1.01‒1.22)	
70–74	1.47 (1.29‒1.67)		1.23 (1.12‒1.34)	
75–79	1.55 (1.36–1.77)		1.28 (1.15–1.41)	
80+	1.63 (1.40–1.90)		1.29 (1.13–1.46)	
Chronic health problems^†^				
Cardiovascular disorders	1.42 (1.28‒1.58)	**<0.001**	1.23 (1.14‒1.33)	**<0.001**
Endocrine disorders	1.48 (1.38‒1.58)	**<0.001**	1.47 (1.31–1.64)	**<0.001**
Bone and joint disorders	1.26 (1.17‒1.35)	**<0.001**	_	
Urologic disorders	1.12 (1.03‒1.21)	**0.006**	0.91 (0.86–0.97)	**0.002**
Ophthalmic disorders	1.15 (1.06‒1.25)	**0.001**	‒	
Respiratory disorders	1.13 (1.00‒1.28)	0.051	_	
Oncologic disorders	1.12 (1.03‒1.32)	**0.014**	_	
Gastrointestinal disorders	1.48 (1.29‒1.68)	**<0.001**	1.13 (1.03–1.24)	**0.010**
Renal disorders	1.59 (1.36‒1.85)	**<0.001**	‒	
Neurologic disorders	1.31 (0.97‒1.77)	0.077	‒	
Psychiatric disorders	1.08 (0.71‒1.64)	0.714	‒	
Smoke cigarettes		**0.002**		
No (reference)	1.00	‒	_	
Current smoker	0.86 (0.76‒0.97)	0.013	_	
Ex-smoker	1.125 (1.01‒1.23)	0.031	_	
Payment method for healthcare service in last 12 months^†^				
Used Free Service	1.14 1.01–1.28)	**0.044**	‒	
Self/Out of pocket	0.90 (0.84‒0.97)	**0.004**	‒	
Employer	1.14 (1.05‒1.23)	**0.001**	‒	
Relative	1.09(0.98‒1.23)	0.123	‒	
Welfare	1.14 (1.02‒1.28)	**0.024**	_	
Insurance	0.73 (0.55‒0.97)	**0.028**	_	
Non-governmental/ religious organization	3.59 (0.37‒34.56	0.270	_	
Alcohol consumption history		**0.01**		
No (reference)	1.00	‒		
Yes	1.10 (1.02‒1.19)	0.013	-	
Used to	1.17 (1.01‒1.35)	0.037		
Employment^†^	0.88 (0.81‒0.97)	**0.009**	‒	
Highest education level		**<0.001**		
Primary or below	1.00	_	_	
Secondary	1.06(0.98–1.15)	0.170	_	
Tertiary	1.22 (1.12–1.33)	<0.001	_	
Gender				
Male	1.06 (0.99‒1.14)	0.085	1.09 (1.03–1.14)	**0.001**

IRR:incidence rate ratio; CI: confidence interval

†”No” is the reference group

Multicollinearity Overdispersion and interaction terms were checked and not found.

Interactions were between the number of supplements and the number of comorbidities and the presence of endocrine disorders and number of comorbidities

Area under the receiver operating characteristic (ROC) curve (79.9%) were applied to check the goodness of qfit

### Health outcomes associated with polypharmacy

Multivariate logistic analysis found that PIMs (OR 1.27; 95% CI: 1.20, 1.34, *p*<0.001) and PDDIs (OR 1.34; 95% CI: 1.26, 1.42, *p*<0.001) were significantly associated with the increase in number of medications used after adjusting for age, gender, ethnicity and the number of comorbidities but not whether falls occurred in the past 12 months or functional impairment existed (Table 3). Multivariate linear regression models found a significant association between the increase in number of medications used and healthcare utilisation (β = 0.44; 95% CI: 0.30, 0.59, *p* <0.001) but not the quality of life scores.

**Table 3:** Health outcomes associated with polypharmacy among urban-community dwelling older adults.

**Health outcomes associated with polypharmacy**				
**Categorical variables**	**Crude OR (95%CI)**	***p-value***	**^**†**^Adjusted OR (95%CI)**	***p-value***
PIMs*	1.31 (1.25‒1.37)	<0.001	1.27 (1.20‒1.34)	<0.001
PDDIs*	1.38 (1.31‒1.46)	<0.001	1.34 (1.26‒1.42)	<0.001
Fall in last 12 months	1.04 (0.99‒1.09)	0.060	‒	‒
Katz ADL	1.04 (0.94‒1.14)	0.473	‒	‒
Lawton IADL	1.09 (1.05‒1.14)	<0.001	‒	‒
**Continuous variables**	**Crude β (95%CI)**	***p-value***	**^**†**^Adjusted β (95%CI)**	***p-value***
Healthcare utilisation	0.46 (0.32–0.60)	<0.001	0.44 (0.30–0.59)	<0.001
CASP 12 QoL	-0.11 (-2.08 -,0.01)	0.040	-	-

OR: odd ratio; CI: confidence interval; PIMs: potential inappropriate medications; PDDIs: potential drug-drug interactions; ADL: activities of daily living; IADL: instrumental activities of daily living

†Adjusted for age, gender, number of comorbidities and ethnicity

Healthcare utilisation unadjusted model:, R2 = 0.003; adjusted model: R2 = 0.038

CASP 12 QoL unadjusted model:, R2 = 0.003;

β: regression coefficient; CI: confidence interval; R2: coefficient of determination; CASP 12 QoL: control, autonomy, self realisation and pleasure measure of quality of life

In a similar analysis performed for prescription/non-prescription drugs and dietary supplements separately, it was found that increased supplement use remained significantly associated with PDDIs but quality of life scores also improved (data not shown). The health impact associated with prescription/non-prescription polypharmacy were similar to that found when the total medication used was analysed.

## Discussion

This study assessed the prevalence, risk factors and health outcomes associated with polypharmacy, including the contributory role of dietary supplement use among older individuals taking chronic medications in the community setting in Malaysia. The study found a high prevalence of polypharmacy (46%) with dietary supplements constituting a third of the total number of medications used in older individuals. Risk factors independently associated with increased medication use were advanced age, being male, increasing number of comorbidities specifically cardiovascular, endocrine, and gastrointestinal disorders, and dietary supplement use. As for ethnic groups, Chinese and Malays were associated with lower risk of polypharmacy compared to Indians as well as individuals diagnosed with urologic disorders. The health outcomes associated with increased medication use were potential inappropriate medication use (PIMs), potential drug-drug interactions (PDDIs) and increased healthcare utilisation while increased medication use was not found to be associated with falls, functional disability and quality of life in this cohort. Increased use of dietary supplement was independently associated with increased PDDIs and improved quality of life scores after adjusting for age, gender, ethnicity and number of comorbidities.

The prevalence of polypharmacy in older individuals varied between 39% and 89% in the Asian regions, depending on the definition of polypharmacy used, the age range of the participants and the research settings [28, 35–39]. Prior studies on polypharmacy among the Malaysian elderly people, reported rates between 39% and 55% in the hospital setting [17, 19] but currently, no data is available for community dwelling individuals. Studies assessing polypharmacy in the community are logistically harder to conduct and reports from other countries often utilized data from health insurance claims [40] or telephone interviews [41] to estimate the rates of polypharmacy in the community. These approaches may be inaccurate due to underreporting and the omission of out of pocket purchases of non-prescription drugs including dietary supplements. In the present study, medication use was reported by the study participants and verified against the actual medications taken and prescriptions during home visits which provided a more accurate estimate of the total medications taken. Notably, 1 in 3 older individuals who were taking chronic medications in the community setting used five or more medications concurrently, with 1 in 2 reporting dietary supplement use. Additionally, more than 30% of the cohort experienced PIMs similar to a prior study among elderly nursing home residents in Malaysia [42] while one fifth were exposed to clinically significant PDDIs. These data highlight high rates of medication related issues among the community-dwelling older individuals in Malaysia, which currently does not have a national programme for medication review in the elderly population, unlike many other countries [43–45]. Given that Malaysia will experience a sizable increase in their older population over the next decade, the introduction of such a programme is timely and the findings from this study would provide baseline data to monitor the changes over time.

Among the risk factors associated with polypharmacy, advanced age and increasing number of comorbidities were associated with increased medication use, similar to that reported in other studies [17, 36, 46, 47]. Specifically, the diagnoses with cardiovascular, endocrine or gastrointestinal disorders were associated with a significant increase in medication use compared to other disease conditions as previously described [17, 38, 46, 48]. Prior studies in the general population in Malaysia which assessed medication related issues in individuals diagnosed with cardiovascular and endocrine disorders had similarly highlighted a profile of multiple concurrent medication use in the management of these conditions [49, 50]. In hypertension and diabetes, which are among the most common cardiovascular and endocrine disorders in older people, numerous studies had found that the overzealous prescribing of medications to reach specific treatment targets may have only marginal benefits compared to the harms associated with hypoglycemia and hypotension in this age group. These may lead to falls, fractures and head injuries which could further impact physical function and quality of life [51–54]. Therefore, many guidelines including those in Malaysia recommend a less intensified approach for blood glucose and blood pressure management among the elderly patients [55–58] though this may not be widely practised [59]. Additionally, Malaysia does not have a national health insurance scheme and thus patients are not managed by a single medical practitioner but are free to visit any medical establishment to seek treatment. This system encourages multi-clinic visits and over-prescription [60] which invariably leads to polypharmacy. The lack of separation between prescribing and dispensing roles between doctors and pharmacists in Malaysia further complicates this situation.

This study found ethnic groups, Malay and Chinese were less likely to be associated with polypharmacy compared to the Indians when all other covariates were held constant. The reasons for this are not entirely clear but polypharmacy disparities among different ethnicities have been previously reported [47, 61] and may be associated with different underlying socio-economic status [47, 62]. The present study did not have detailed information on the socio-economic status of the study participants but similar to a prior study [62], the univariate analysis showed that individuals paying from health insurance or paying out of pocket for health services were less likely to experience polypharmacy compared to individuals receiving free or subsidised healthcare services. Future studies should explore the interaction between ethnicity and socio-economic status on polypharmacy and medication related issues in Malaysia.

In the multivariate analysis, an increase in the use of dietary supplements was associated with almost double the incident risk ratio of polypharmacy (15%) compared to the increase in the number of comorbidities (8%). This highlights the important contributory role of supplement use to polypharmacy in the present study setting. A market survey on the profile of regular supplement users in Malaysia found older individuals often considered taking dietary supplements as ‘nutritional insurance’ because they were concerned about their health following the diagnosis of chronic conditions [63]. More than two-thirds of the participants who reported using dietary supplement in the present study, had two or more comorbidities. Supplement use was also highest among elderly individuals diagnosed with oncologic disorders, as previously reported [64–66] though scientific evidence on the safety and efficacy of supplements in cancer has so far been inconclusive (reviewed in [67]). In the present study, a significant association between supplement use and potential drug-drug interactions was found and this remained significant after adjusting for age, gender, ethnicity and multiple comorbidities. Thus, the high use of supplements among elderly people is a major concern, particularly given that a majority of patients in our setting have previously reported not to disclose information on their supplement intake to their healthcare practitioners [64, 65].

Association between supplement use and quality of life scores has not been consistent in the literature. Some studies reported a positive association [68–70] while others reported a negative impact on quality of life [71], depending on the disease setting. In the present study, an increase in supplement use in the elderly was associated with better quality of life scores. It is important to note that the CASP-12 tool used to measure QoL in this study only encompassed the psychosocial constructs of quality of life including domains of control, autonomy, pleasure and self-realization but not physical aspects. However, no association was found between an increase in supplement use and measures of physical functioning including ADL and IADL. Therefore, the association between supplement use and improved quality of life should be interpreted with caution as other factors including socio-economic status which could also impact QoL were not assessed and adjusted in the present model.

Thus far, few studies have assessed health outcomes associated with polypharmacy in the community dwelling elderly (reviewed in [8]). In the multivariate analysis, it was found that increasing medication use (prescribed, non-prescribed and supplement use) was associated with PDDIs and PIMS after adjusting for age, sex, ethnicity and the number of comorbidities, as previously described [9, 72, 73]. In the present study, only drug interactions which were considered clinically significant and could potentially lead to serious consequences were assessed while PIMS identified the use of medications that had a high propensity to cause adverse events in the elderly population. Both of these outcomes have significant morbidity and mortality implications in the elderly population and are frequent causes of preventable medication related hospitalisations [54, 74, 75]. In addition, it was found that increasing medication use was independently associated with increased healthcare visits to any healthcare facility in the prior 12 months though the reasons for these visits were not explored in this study. The use of multiple drugs is sometimes unavoidable in individuals with multiple comorbidities. However, this needs to be weighed against the potential harms of increasing treatment burden in the elderly population. The findings in the present study highlight an urgent need to introduce medication reconciliation programmes among community-dwelling elderly population and increase educational initiatives among healthcare practitioners with regards to prescribing issues in older individuals. Approaches which have been shown to reduce adverse drug events and hospitalisation associated with polypharmacy should be considered [76–79]. Polypharmacy was not associated with falls, physical function (measured by ADL and IADL) nor quality of life scores in this study though these associations were significant in the univariate model and a model which did not include comorbidities as a covariate. This implies that the presence of multiple comorbidities has a stronger modulating effect on these outcomes. Additionally, recent studies including those in the present study setting found that the consumption of falls-risk increasing drugs, rather than polypharmacy per say were more important in predicting the risk of falls in the elderly population [18, 80].

There were a number of limitations in the present study. Firstly, participants’ adherence to the medications in their possession was not assessed and this could have affected the other variables. Secondly, there were some intrinsic limitations to the use of the 2015 Beers criteria to assess PIMS as many of the medications listed in Beers were not available in the present study setting. The Beers criteria was used instead of STOPP/START, the other commonly used tool to measure PIMS in the elderly population. This was because the participants’ medical notes were not available and hence, assessing the appropriateness of medications prescribed as required by the latter tool could not be conducted. The present study could not exclude the possibility that safer alternatives to the inappropriate medications listed in Beers had not already been tried in the past or that it was the participants’ choice to continue using these medications despite the inherent risks. The strengths of the present study include the study approach which involved home visits that allowed a realistic estimate of the number of medications taken by the participants. In addition, the use of dietary supplements could be documented in the cohort as this is often omitted in studies which assessed polypharmacy. The sample size was large and representative of the urban community setting in Malaysia.

## Conclusions

A number of significant health related issues specifically, PIMS, PDDIs and increased healthcare utilisation were found to be associated with polypharmacy among older urban community dwelling individuals who were taking chronic medications. Dietary supplement use was also high among older individuals and was significantly associated with PDDIs. Individuals diagnosed with cardiovascular, endocrine and gastrointestinal disorders had a high propensity to receive multiple medications which contributed significantly to polypharmacy. The present study adds to the growing evidence that a significant proportion of older adults are exposed to multiple medication related issues. More stringent guidelines with regards to the appropriateness and therapeutic need of dietary supplements among older adults is also crucial, given its contribution to polypharmacy in the present study. Healthcare providers should be vigilant of such usage to prevent any interactions with prescribed medications. Pharmacists, especially those in the community setting should provide targeted medication reviews for older individuals despite the lack of a national programme to reduce the potential morbidity associated with polypharmacy in this age group.

## Supporting information

S1 File Supplementary data 1: Assumptions made during assessments of potential inappropriate medications (PIMs) use according to the American Geriatrics Society 2015 Updated Beers Criteria. (PDF)

S2 File Supplementary data 3: Relevant sections of the questionnaire used for the study (English and Malay versions). (PDF)

S3 File Supplementary data 4: Minimal dataset for the study. (XLS)

S1 Table Supplementary Table 1: Prevalence of medication classes used according to the Anatomical Therapeutic Chemical (ATC) Classification System (ATC first level) among 1256 urban-community dwelling older adults. (PDF)

S2 Table Supplementary Table 2. Details of the products taken by the 715 dietary supplements users.
(PDF)
