# Supplementary material for: Prevalence, risk factors and health outcomes associated with polypharmacy among urban community-dwelling older adults in multi-ethnic Malaysia
Source: PLoS One. 2017 Mar 8;12(3):e0173466. doi: 10.1371/journal.pone.0173466 (PMC5342241; doi:10.1371/journal.pone.0173466)
Supplement: S1 File — (PDF) [file pone.0173466.s001.pdf]

Supplementary data 1: Assumptions made during assessments of potential inappropriate medications (PIMs) use according to the American Geriatrics Society 2015 Updated Beers Criteria

1. Missing doses were assumed to be within the dose range suggested.
2. History of peptic ulcer was assessed using a reported history of gastroesophageal reflux/peptic ulcer in the questionnaire.
3. Syncope syndrome was assessed using self-reported episodes of blackout in the last 12 months in the questionnaire.
4. History of fall or fracture was assessed using self-reported history of fall in last 12 months or history of fracture in past lifetime. When responses were missing (in 1.4% of the cohort) they were assumed to have not experienced falls or fractures.
5. Self-reported chronic kidney disease/kidney failure was used to replace data on renal function when creatinine clearance was not recorded (n=524/1256 or 42% of individuals)
6. Participants using antiplatelet, anticoagulant and having peptic ulcer were assumed to be high-risk patients and rationalized proton pump inhibitor use.
7. The risk of delirium could not be assessed.
