# Supplementary material for: Prevalence, risk factors and health outcomes associated with polypharmacy among urban community-dwelling older adults in multi-ethnic Malaysia
Source: PLoS One. 2017 Mar 8;12(3):e0173466. doi: 10.1371/journal.pone.0173466 (PMC5342241; doi:10.1371/journal.pone.0173466)
Supplement: S2 File — (PDF) [file pone.0173466.s002.pdf]

MELoR

MALAYSIAN ELDERS LONGITUDINAL RESEARCH

GLOBAL ASSESSMENT QUESTIONNAIRE
(EDITTED SECTIONS A B E H I J K L)
ENGLISH VERSION (PAGE 1-26)
MALAY VERSION (PAGE 28-51)

PART A: INFORMATION

A.1 INTERVIEWER'S INFORMATION

1. IC Number
☐☐☐☐☐☐-☐☐-☐☐☐☐

2. Name
☐☐☐☐☐☐☐☐☐☐☐☐☐☐☐☐☐☐☐☐☐
☐☐☐☐☐☐☐☐☐☐☐☐☐☐☐☐☐☐☐☐☐

3. Interview date
☐☐ /☐☐ /☐☐☐☐ (dd/mm/yy)

4. Interview time
☐☐:☐☐ (am/pm)

5. Gender
☐1= Male
☐2= Female

A.2 RESPONDENT'S INFORMATION

1. IC Number
□□□□□□-□□-□□□□

2. Name
□□□□□□□□□□□□□□□□□□□□□
□□□□□□□□□□□□□□□□□□□□□

3. Address
□□□□□□□□□□□□□□□□□□□□□
□□□□□□□□□□□□□□□□□□□□□
□□□□□□□□□□□□□□□□□□□□□
□□□□□□□□□□□□□□□□□□□□□

4. District
□□□□□□□□□□□□□□□□□□□□□

5. City
□□□□□□□□□□□□□□□□□□□□□

6. State
□□□□□□□□□

7. Postcode
□□□□□

8. Telephone
(H) □□-□□□□□□□□
(M) □□□-□□□□□□□□□

\

A.3 ALTERNATIVE CONTACT

1. Name

2. Relationship

3. Telephone

(H) -

(M) -

PART B: SOCIO-DEMOGRAPHIC INFORMATION

B.1 PERSONAL DESCRIPTION

1. Sex
   - ☐ 1= Male
   - ☐ 2= Female

2. Ethnicity
   (Based on father's ethnicity)
   - ☐ 1= Malay
   - ☐ 2= Chinese
   - ☐ 3= Indian
   - ☐ 4= Other Bumiputra
   - ☐ 5= Others
   - ☐ 99=Not Related

3. Religion:
   - ☐ 1= Islam
   - ☐ 2= Christian
   - ☐ 3= Buddhist
   - ☐ 4= Hindu
   - ☐ 5= Sikh
   - ☐ 6= Others
   - ☐ 7= Atheist
   - ☐ 88= Don't Know
   - ☐ 99= Not Related

B.4 EDUCATIONAL LEVEL

1. Highest education level:
   - ☐ 1= No formal education
   - ☐ 2= Primary
   - ☐ 3= Secondary
   - ☐ 4= Certificate/Skill (Post-Secondary)
   - ☐ 5= College/University
   - ☐ 99= Not Related

PART E: SOCIAL PARTICIPATION

E.1 VOCATIONAL INFORMATION

1. Have you ever worked?
(Boolean)
☐ 1= No **(Go to Q4)**
☐ 2= Yes

2. Are you still working?
(Boolean)
☐ 1= No **(Go to Q3)**
☐ 2= Yes

PART H: FUNCTIONAL & PHYSICAL ACTIVITIES

H.1 KATZ

No	Can you do these activities?	1	2	3
		No	Yes	Yes, but with some help
1	Walk across a small room?	☐	☐	☐
2	Bathing, either a sponge bath, tub bath or shower?	☐	☐	☐
3	Personal grooming, like brushing hair, brushing teeth, or washing face?	☐	☐	☐
4	Dressing, like putting on a shirt, buttoning and zipping, or putting on shoes?	☐	☐	☐
5	Eating like holding a fork, cutting food, or drinking from a glass?	☐	☐	☐
6	Getting from a bed to a chair?	☐	☐	☐
7	Using the toilet?	☐	☐	☐

H.2 INSTRUMENTAL ACTIVITIES OF DAILY LIVING

Do you currently have difficulty carrying out any of the following activities on your own? Please TICK 'Yes' or 'No' if this is a result of a long term health or medical problems, or due to old age?

1. Using the telephone?
   ☐1= Without help including looking up numbers and dialling
   ☐2= With some help (can answer phone or dial operator in an emergency, but need a special phone or help in getting the number or dialling)
   ☐3= Or are you completely unable to use the telephone

2. Get to places out of walking distance?
   ☐1= Without help (can travel alone on busses, taxis or drive your own car)
   ☐2= With some help (need someone to help you or go with you when travelling)
   ☐3= Or are you unable to travel unless emergency arrangements are made for a specialized vehicle like and ambulance

3. Shopping for groceries or clothes?
   ☐1= Without help (taking care of all shopping needs yourself, assuming you had transportation)
   ☐2= With some help (need someone to help you or go with you on all shopping trips)
   ☐3= Or are you unable to do any shopping

4. Preparing your own meal?
   ☐1= Without help, (plan or cook full meals for yourself)
   ☐2= With some help (can prepare some things but unable to cook full meals yourself)
   ☐3= Or are you completely unable to prepare any meals?

☐4= I can do this but I don’t need to eg.I have a maid or carer that does this for me

5. Doing house work on your own?
   ☐1= Without help, (can scrub floors, etc)
   ☐2= With some help (can do light housework but need help with heavy work)
   ☐3= Or are you completely unable to do any housework?
   ☐4= I can do this but I don’t need to eg.I have a maid or carer that does this for me

6. Taking your own medication?
   ☐1= Without help (in the right doses at the right time)
   ☐2= With some help (are able to take medications if someone prepares it for you and/ or reminds you to take it)
   ☐3= Or are you completely unable to take your own medication?
   ☐4= Non-applicable (N/A)

7. Handle your own money?
   ☐1= Without help (able to pay bills, write cheques)
   ☐2= With some help (manage day-to-day purchases but need help with managing your cheque book and paying your bills)
   ☐3= Or are you completely unable to handle money?

8. Doing heavy work around the house? (i.e. washing windows, etc)
   ☐1= No
   ☐2= Yes

9. Walk up and down the stairs?
   ☐1= No
   ☐2= Yes

10. Walk a mile (without help)
   ☐1= No
   ☐2= Yes

11. Drive a car or other vehicle?
   ☐1= Without help (I have no problem to get where I want to go)
   ☐2= With some help (I am not confident e.g driving to unfamiliar places, need someone with me or etc)
   ☐3= I am completely unable to drive currently
   ☐4= Non-applicable (N/A)

H.3 INTERNATIONAL PHYSICAL ACTIVITY QUESTIONNAIRE – IPAQ

1. During the last 7 days, on how many days did you do vigorous physical activities like heavy lifting,
digging, aerobics, or fast bicycling?
☐ 1= No vigorous physical activity **(Go to Q3)**
☐_____2= Enter number of days per week of vigorous activity

2. How much time did you usually spend doing vigorous physical activities on one of those days?
☐ 1= Don't know/Not Sure
☐_____2= Enter Hours/Minutes per day

3. During the last 7 days, on how many days did you do moderate physical activities like carrying light loads, bicycling at a regular pace, or doubles tennis? Do not include walking.
☐ 1= No moderate physical activity **(Go to Q5)**
☐_____2= Enter number of days per week of moderate physical activity

4. How much time did you usually spend doing moderate physical activities on one of those days?
☐ 1= Don't know/Not Sure
☐_____2= Enter Hours/Minutes per day

5. During the last 7 days, on how many days did you walk for at least 10 minutes at a time?
☐ 1= No walking activity **(Go to Q7)**
☐_____2= Enter number of days per week of 10 minutes walking

6. How much time did you usually spend walking on one of those days?
☐ 1= Don't know/Not Sure
☐_____2= Enter Hours/Minutes per day

7. During the last 7 days, how much time did you spend sitting on a week day?
☐ 1= Don't know/Not Sure
☐_____2= Enter Hours/Minutes per day

I.2. FALLS QUESTIONNAIRE

(Interviewer- READ OUT- I would like to ask you few questions whether you have fallen in the past)

1. Have you fallen in the past 12 months?
   - ☐ 1= No (move on to Question 2)
   - ☐ 2= Yes
   - ☐ 3= Don’t know (move on to Question 2)

PART J: QUALITY OF LIFE

J.1 CASP-19

Interviewer READ OUT: "Here is a list of statements that people have used to describe their lives or how they feel. We would like to know how often, if at all, you think this applies to you"

		1	2	3	4
		**Often**	**Sometimes**	**Not often**	**Never**
i.	My age prevents me from doing the things I would like to	☐	☐	☐	☐
ii.	I feel that what happens to me is out of my control	☐	☐	☐	☐
iii.	I feel free to plan for the future	☐	☐	☐	☐
iv.	I feel left out of things	☐	☐	☐	☐
v.	I can do the things that I want to do	☐	☐	☐	☐
vi.	Family responsibilities prevent me from doing what I want to do	☐	☐	☐	☐
vii.	I feel that I can please myself what I do	☐	☐	☐	☐

		1	2	3	4
		**Often**	**Sometimes**	**Not often**	**Never**
viii.	My health stops me from doing things I want to do	☐	☐	☐	☐
ix.	Shortage of money stops me from doing things I want to do	☐	☐	☐	☐
x.	I look forward to each day	☐	☐	☐	☐
xi.	I feel that my life has meaning	☐	☐	☐	☐
xii.	I enjoy the things that I do	☐	☐	☐	☐
xiii.	I enjoy being in the company of others	☐	☐	☐	☐
xiv.	On balance, I look back on my life with a sense of happiness	☐	☐	☐	☐
xv.	I feel full of energy these days	☐	☐	☐	☐
xvi.	I choose to do things that I have never done before	☐	☐	☐	☐
xvii.	I feel satisfied with the way my life has turned out	☐	☐	☐	☐
xviii.	I feel that life is full of opportunities	☐	☐	☐	☐
xix.	I feel that the future looks good for me	☐	☐	☐	☐

PART K: GENERAL HEALTH AND MEDICAL HISTORY

K.1 GENERAL HEALTH

1. Would you say your health is?
   - ☐1= Poor
   - ☐2= Fair
   - ☐3= Good
   - ☐4= Very good
   - ☐5= Excellent
   - ☐99= Don't Know

2. In general, compared to other people of your age, would you say your health is….
   - ☐1= Poor
   - ☐2= Fair
   - ☐3= Good
   - ☐4= Very good
   - ☐5= Excellent
   - ☐99= Don't Know

3. Is your present state of health causing problems with any of the following?

3.1 Job (paid employment)	1= No ☐	2= Yes ☐
3.2 Household chores	1= No ☐	2= Yes ☐
3.3 Social life	1= No ☐	2= Yes ☐
3.4 Sex life	1= No ☐	2= Yes ☐
3.5 Interests and hobbies	1= No ☐	2= Yes ☐
3.6 Holidays and outings	1= No ☐	2= Yes ☐
3.7 Family relationships	1= No ☐	2= Yes ☐

4. In the past year have you lost 10 pounds (4.5 kg) or more in weight when you weren't trying to, for example, because of illness?
   - ☐1= No
   - ☐2= Yes
   - ☐66= Refuse to Answer
   - ☐99= Don't Know

K.2 VISION

1. Do you wear glasses or contact lenses?
   (Interviewer: Don't ask if the participant is wearing glasses)
   - ☐1= No **(Go to Q 2)**
   - ☐2= Yes

   1.1 Which type of glasses/contact lenses?
   - ☐1= Distance glasses
   - ☐2= Reading glasses
   - ☐3= Bifocals
   - ☐4= Multifocal

2. Is your eyesight (using glasses or contact lens, if you use them)...
   (Interviewer: READ OUT OPTIONS)
   - ☐1= Excellent
   - ☐2= Very Good
   - ☐3= Good
   - ☐4= Fair
   - ☐5= Poor
   - ☐6= Registered or Legally Blind
   - ☐66= Refuse To Answer
   - ☐99= Don't Know

3. How good is your eyesight for seeing things at a distance, like recognizing a friend across the street (using glasses or contact lens if you use them)? Would you say it is…
   (Interviewer: READ OUT OPTIONS)
   - ☐1= excellent
   - ☐2= very good
   - ☐3= good
   - ☐4= fair
   - ☐5= Poor
   - ☐66= Refuse to Answer
   - ☐99= Don't Know

4. Has a doctor ever told you that you have any of the following eye diseases?
   (Interviewer: READ OUT OPTIONS)
   - ☐1= None (no eye disease)
   - ☐2= Glaucoma
   - ☐3= Age related macular degeneration
   - ☐4= Other
   - ☐5= Cataracts **(Go to Q5)**
   - ☐66= Refuse to answer
   - ☐88= Don't Know

5. Have you had cataract surgery?

☐1= No

☐2= One eye

☐5= Both eyes

☐66= Refuse to answer

☐88= Don’t Know

K.3 HEARING

1. Do have any problems with your hearing?
   - ☐1= No (Go to 1.3)
   - ☐2= Yes (Go to 1.1)

   1.1 Do you use a hearing aid?
   - ☐1= No (Go to 1.2)
   - ☐2= Yes (Go to 1.3)

   1.2 If you are not wearing a hearing aid, please state why
   - ☐1= No reason
   - ☐2= I don't feel that I need it
   - ☐3= It is too expensive
   - ☐4= I find it difficult to wear
   - ☐5= I don't like using it
   - ☐6= Other

   1.3 Is your hearing (for those with or without a hearing aid)
   (Interviewer: READ OUT OPTIONS)
   - ☐1= Excellent
   - ☐2= Very good
   - ☐3= Good
   - ☐4= Fair
   - ☐5= Poor
   - ☐66= Refuse to answer
   - ☐88= Don't know

K.4 MEMORY

1. How would you rate your day-to-day memory at the present time? Would you say it is.......
   (**Interviewer: READ OUT OPTIONS)**
   - ☐1= Excellent
   - ☐2= Very good
   - ☐3= Good
   - ☐4= Fair
   - ☐5= Poor
   - ☐66= Refuse to answer
   - ☐88= Don't Know

1. How often would you find that you are absent minded, for example forgetting where you put your glasses/keys or finding yourself in a room having forgotten why you came in there? Would you say that you are absent minded in this sort of way?
   (Interviewer: READ OUT OPTIONS)
   - ☐1= All of the time
   - ☐2= Most of the time
   - ☐3= Some of the time
   - ☐4= None of the time
   - ☐66= Refuse to answer
   - ☐88= Don't Know

2. Have you ever been told by family, friends or relatives that your memory is poor or that you are forgetful/absent-minded?
   (Interviewer: READ OUT OPTIONS)
   - ☐1= All of the time
   - ☐2= Most of the time
   - ☐3= Some of the time
   - ☐4= None of the time
   - ☐66= Refuse to answer
   - ☐88= Don't Know

K.5 MEDICAL HISTORY

Has a doctor ever told you that you have any of the following conditions? (Interviewer: READ OUT)

**1.**	**Heart disease**	**No (1)**	**In last 1 year (2)**	**More than 1 year ago(3)**	**Don't Know (4)**
	a. Heart attack	☐	☐	☐	☐
	b. Angina	☐	☐	☐	☐
	c. An Irregular heart rhythm	☐	☐	☐	☐
	d. High blood pressure	☐	☐	☐	☐
	e. High Cholesterol	☐	☐	☐	☐
	f. Cardiac Arrest (Heart stopped and needing resuscitation)	☐	☐	☐	☐
	g. Congestive heart failure	☐	☐	☐	☐
	f. Heart procedure (eg. Angioplasty / endarterectomy / stent)	☐	☐	☐	☐
	g. Cardiac bypass (open heart surgery)	☐	☐	☐	☐
	h. Pacemaker	☐	☐	☐	☐
	i. Any other heart problem (Please Specify____________________)	☐	☐	☐	☐
**2.**	**Heart disease symptoms/signs**				
	a. Chest pain lasting >30 minutes	☐	☐	☐	☐
	b. Any pain or discomfort in your chest?	☐	☐	☐	☐
	c. Palpitations (awareness of your heart beating)	☐	☐	☐	☐
**3.**	**Family history (immediate family) of cardiovascular* disease or sudden death from cardiovascular* causes** *includes cardiac, stroke or other vascular disease	☐ **No**	☐ **Yes**		

Has a doctor ever told you that you have any of the following conditions? (Interviewer: READ OUT)

4.	Respiratory Diseases	No (1)	In last 1 year (2)	More than 1 year ago (3)	Don't Know (4)
	a. Asthma	☐	☐	☐	☐
	b. Bronchitis	☐	☐	☐	☐
	c. Emphysema/COPD - Chronic Obstructive Pulmonary Disease	☐	☐	☐	☐
5.	**Respiratory Symptoms/Signs**				
	a. Shortness of breath (when walking on level ground)	☐	☐	☐	☐
	b. Sputum production in the morning	☐	☐	☐	☐
	c. Cough or increased sputum production for >3 months over past 4 years	☐	☐	☐	☐

6.	Cerebrovascular disease	No (1)	In last 1 year (2)	More than 1 year ago (3)	Don't Know (4)
	a. Stroke (see Appendix)	☐	☐	☐	☐
	If this occurred state year:		__ __ __ __	__ __ __ __	
	**a.1. If yes, how many strokes did you have?**		________ (No. of Stroke cases)		
	b. Transient ischemic attack* (mini stroke/TIA) **(Stroke symptoms* that last a few minutes to 24 hours)**	☐	☐	☐	☐
	If this occurred state year:		__ __ __ __	__ __ __ __	
	**b. 1 If yes, how many TIAs did you have?**		________ (No. of TIA cases)		

7.	Other neurologic conditions	No (1)	In last 1 year (2)	More than 1 year ago (3)	Don't Know (4)
	a. Seizures/fits (including epilepsy)	☐	☐	☐	☐

	b. Traumatic brain injury (due to head trauma)	☐	☐	☐	☐
	c. Dementia<br>**Specify type (If known):________**	☐	☐	☐	☐
	d. Parkinson's disease	☐	☐	☐	☐
	d. Other (*specify*)<br>________________________________<br>(e.g., Peripheral neuropathy)	☐	☐	☐	☐

8.	Malignancy	No (1)	Within last 1 year (2)	More than 1 year ago (3)	Don't Know (4)
	a. Cancer	☐	☐	☐	☐
	b**. If YES,**				
	i. Breast	☐	☐	☐	☐
	ii. Lung	☐	☐	☐	☐
	iii. Bowel	☐	☐	☐	☐
	iv. nasopharyngeal	☐	☐	☐	☐
	v. Other (specify)<br>________________________________	☐	☐	☐	☐
**9.**	**Other health conditions**	**No (1)**	**In last 1 year (2)**	**More than 1 year ago (3)**	**Don't Know (4)**
	a. Diabetes	☐	☐	☐	☐
	b. Vitamin B12 deficiency	☐	☐	☐	☐
		☐	☐	☐	☐

c. Intermittent claudication (pain in legs when walking due to blockage in arteries)				
d. Chronic kidney disease or failure	☐	☐	☐	☐
e. Thyroid disease (Underactive or hyperactive)	☐	☐	☐	☐
f. Indigestion (Gastroesophageal Reflux/Stomach Ulcer/Duodenal Ulcer)	☐	☐	☐	☐
g. Liver disease (Hepatitis B/C/D/E/Cirrhosis)	☐	☐	☐	☐
h. Arthritis (Osteoarthritis/Rheumatoid Arthritis/Others:_____________)	☐	☐	☐	☐
i. Osteoporosis, sometimes called thin or brittle bones	☐	☐	☐	☐
j. Gout (high uric acid)	☐	☐	☐	☐
k. Depression	☐	☐	☐	☐
l. Anxiety	☐	☐	☐	☐
m. Other psychiatric conditions (specify)___________	☐	☐	☐	☐
**n. Incontinence**				
Urine:				
i. Do you ever wet yourself when you cough or strain?	☐	☐	☐	☐
ii. Do you ever wet yourself before you reach the toilet?	☐	☐	☐	☐
o. Others, please list: (please add another sheet if required)				

______________________________	☐	☐	☐	☐
______________________________	☐	☐	☐	☐
______________________________	☐	☐	☐	☐

10. History on Substance

a. Do you drink any alcohol?

☐ 1= No ☐ 2= Yes ☐ 3= Never ☐ 4= I used to

i. If yes, would you describe your present alcohol intake as :

☐ 1= Daily/most days ☐ 2= Weekends only ☐ 3= Once or Twice a month ☐ 4= Special occasions

ii. What type of drink do you usually take?

☐ 1= Beers ,lagers
☐ 2= Sherry ,wine
☐ 3= Spirits
☐ 4= Variety of beer, wines or spirits
☐ 5= Low alcohol drinks

iii. One drink is HALF a pint of beer, a SINGLE whisky, gin etc., or ONE GLASS of wine or sherry. How much do you usually drink each day?

☐ 1= More than 6 drinks a day
☐ 2= 3-6 drinks a day
☐ 3= 2 drinks a day or less
☐ 4= None

iv. How many alcoholic drinks do you drink in an average week? __________drinks

v. If you used to drink,

How many years ago did you stop drinking? ☐☐

How many years did you drink? ☐☐

vi. Why did you stop?

____

vii. How many alcoholic drinks did you drink in an average week? __________drinks

b. Smoking history

Do you smoke cigarettes?	☐ 1= No	☐ 2= Yes	☐ 3= Never	☐ 4= Ex-smoker
i. If yes, number of cigarettes smoked daily	☐☐			
ii. If ex-smoker; at what age did you stop?	☐☐		Total years of smoking	☐☐
Amount of cigarette smoked/day	☐☐			
iii. Does your partner smoke cigarettes?	☐ 1= No	☐ 2= Yes	☐ 3= Unknown	☐ 4= Ex-smoker
iii. If yes; How many a day?	☐☐			
How long has he/she been smoking?	☐☐			
iv. Do you smoke any other substance?	☐ 1= Pipe	☐ 2= Cigar	☐ 77= Other: _____________	

11. MEDICATION HISTORY

INTERVIEWER: READ OUT: Now I would like to record all medications that you take on a regular basis, like every day or every week. This will include prescription and non-prescription medications, over-the-counter (OTC) medicines, vitamins, and herbal and alternative medicines.

INTERVIEWER: ASK RESPONDENT IF YOU COULD SEE THE MEDICATIONS HE/SHE TAKES SO YOU CAN COPY DOWN THE CORRECT SPELLING OF EACH TABLET.

1.	
2.	
3.	
4.	
5.	
6.	
7.	
8.	
9.	
10.	
11.	
12.	
13.	
14.	
15.	

PART L: HEALTHCARE UTILIZATION

L.1 OUTPATIENT CARE (FOR ACUTE ILLNESS OR REGULAR HEALTH CHECK)

1. For any illness you may have had in the past 12 months, did you seek medical care at any of these facilities? State the frequency of visits
   (Multiple choice,Numeric)
   ☐1=None **(Go to 2)** _______
   ☐2=Public hospital _______
   ☐3=Private hospital _______
   ☐4=Public clinic _______
   ☐5=Private clinic _______
   ☐6=Traditional/alternative medical practitioner _______

2. If you did not seek medical care or visit a doctor in the past 12 months, why?
   (Multiple choice)
   ☐1=Not serious enough
   ☐2=No one to bring me
   ☐3=No transport
   ☐4=Too far
   ☐5=Costly (doctor's fee,transport,etc)
   ☐6=Fear of hospital
   ☐7=No one can help me
   ☐8=Self-medication
   ☐77= Others (Please specify ____________)

3. In the last 12 months, if you spent money on healthcare, who paid for it?
   (Multiple choice)
   ☐1=Self
   ☐2=Relatives
   ☐3=Employer
   ☐4=Welfare (Social aid or organization)
   ☐5=NGO or religious organization
   ☐6=Insurance
   ☐66=Refuse to answer
   ☐88=Don’t Know

MALAY version

BAHAGIAN A: MAKLUMAT

A.1 Maklumat Penemubual/ Pembantu Penyelidik

1. No. Kad Pengenalan
   ☐☐☐☐☐☐-☐☐-☐☐☐☐

2. Nama
   ☐☐☐☐☐☐☐☐☐☐☐☐☐☐☐☐☐☐☐☐☐
   ☐☐☐☐☐☐☐☐☐☐☐☐☐☐☐☐☐☐☐☐☐

3. Tarikh Temubual
   ☐☐ /☐☐ /☐☐☐☐ (hb/bln/thn)

4. Masa Temubual
   ☐☐:☐☐ (pg/ptg)

5. Jantina
   ☐1= Lelaki
   ☐2= Perempuan

A.2 MaklumatResponden

1. No. Kad Pengenalan
□□□□□□-□□-□□□□

2. Nama
□□□□□□□□□□□□□□□□□□□□□
□□□□□□□□□□□□□□□□□□□□□

3. Alamat
□□□□□□□□□□□□□□□□□□□□□
□□□□□□□□□□□□□□□□□□□□□
□□□□□□□□□□□□□□□□□□□□□
□□□□□□□□□□□□□□□□□□□□□

4. Daerah
□□□□□□□□□□□□□□□□□□□□□

5. Bandar
□□□□□□□□□□□□□□□□□□□□□

6. Negeri
□□□□□□□□□

7. Poskod
□□□□□

8. No. Telefon
(R) □□-□□□□□□□□
(B) □□□-□□□□□□□□□

A.3 Maklumat Alternatif

1. Nama

2. Hubungan

3. No. Telefon

(R) -

(B) -

BAHAGIAN B: MAKLUMAT DEMOGRAFI SOSIAL

B.1 Maklumat Peribadi

1. Jantina
   - ☐ 1=Lelaki
   - ☐ 2= Perempuan

2. Etnik
   (Berdasarkan etnik sebelah bapa)
   - ☐ 1= Melayu
   - ☐ 2= Cina
   - ☐ 3= India
   - ☐ 4= Lain-lain (Bumiputera)
   - ☐ 5= Lain-lain
   - ☐ 99=Tidak berkaitan

3. Agama:
   - ☐ 1= Islam
   - ☐ 2= Kristian
   - ☐ 3= Buddha
   - ☐ 4= Hindu
   - ☐ 5=Sikh
   - ☐ 6= Lain-lain
   - ☐ 7= Ateis
   - ☐ 88= Tidak tahu
   - ☐ 99=Tidak berkaitan

B.4 TARAF PENDIDIKAN

1. Tahap pendidikan tertinggi:
   - ☐ 1=Tidak bersekolah
   - ☐ 2= Sekolah rendah
   - ☐ 3= Sekolah menengah
   - ☐ 4= Sijil/Kemahiran (Sijil lepasan sekolah menengah)
   - ☐ 5=Kolej/Universiti
   - ☐ 99=Tidak berkaitan

BAHAGIAN E: PENGLIBATAN SOSIAL

E.1 Maklumat Vokasional

1. Adakah anda pernah bekerja?
   (Boolean)
   ☐ 1=Tidak (Sila ke soalan 4)
   ☐ 2=Ya

2. Adakah anda masih bekerja?
   (Boolean)
   ☐ 1=Tidak (Sila ke soalan 3)
   ☐ 2=Ya

BAHAGIAN H: FUNGSI DAN AKTIVITI FIZIKAL

H.1 KATZ

No	Adakah anda boleh melakukan aktiviti ini?	1	2	3
		Tidak	Ya	Ya, tetapi dengan sedikit
1	Berjalan melintasi ruangan/bilik yang kecil?	☐	☐	☐
2	Mandi? (Sama ada menggunakan tab mandian, air pancuran)	☐	☐	☐
3	Perapian peribadi seperti menyisir rambut, memberus gigi atau membersihkan muka?	☐	☐	☐
4	Memakai pakaian seperti memakai baju, mengenakan butang dan zip atau memakai kasut?	☐	☐	☐
5	Makan seperti memegang sudu dan garfu, memotong makanan atau minum dengan menggunakan gelas?	☐	☐	☐
6	Bangun dari tempat tidur untuk duduk di kerusi?	☐	☐	☐
7	Menggunakan tandas?	☐	☐	☐

H.2 AKTIVITI INSTRUMENTAL KEHIDUPAN HARIAN

Adakah anda boleh melakukan aktiviti berikut sendiri? Sila tandakan "Ya" atau "Tidak" jika disebabkan masalah kesihatan, atau disebabkan umur?

1. Menggunakan telefon?
   ☐1= Tidak memerlukan bantuan termasuk mencari nombor telefon dan mendail nombor
   ☐2= Dengan sedikit bantuan (Boleh menjawab telefon dan mendail operatorjika berlaku kecemasan tetapi memerlukan telefon khas atau bantuan orang lain untuk mendapatkan nombor dan mendail)
   ☐3= Atau, adakahanda langsung tidak mampu untuk menggunakan telefon

2. Pergi ke sesuatu tempat diluar jarak berjalan kaki?
   ☐1= Tidak memerlukan bantuan (boleh menaiki bas, teksi atau memandu kenderaan sendiri untuk ke sesuatu tempat)
   ☐2= Dengan sedikit bantuan (memerlukan seseorang untuk membantu atau menemani semasa pergi ke sesuatu tempat
   ☐3=Atau, adakah anda langsungtidak mampupergi ke sesuatu tempatmelainkan dengan bantuan khusus dengan menggunakan kenderaankhas seperti ambulans.

3. Membeli belah untuk barangan dapur dan pakaian?
   ☐1=Tidak memerlukan bantuan (mengambil kira semua barangan keperluan dan mempunyai pengangkutan sendiri)
   ☐2= Dengan sedikit bantuan (memerlukan seseorang untuk menemani anda setiap waktu semasa membeli belah)
   ☐3= Atau, adakah anda langsung tidak mampu untuk melakukan aktiviti membeli-belah

4. Menyediakan makan untuk diri sendiri?

☐1=Tidak memerlukan bantuan (merancang atau memasak semua masakan tanpa bantuan orang lain)

☐2= Dengan sedikit bantuan (boleh menyediakan beberapa jenis masakan tetapi tidak dapat menyediakan masakan tersebut secara keseluruhan)

☐3= Atau, adakah anda langsung tidak mampuuntuk menyediakan makanan

☐4=Saya boleh lakukan tapi saya tidak perlu untuk melaksanakannya (cth. Saya mempunyai orang gaji atau penjaga yang akan melakukannya untuk saya)

5. Melakukan kerja rumah sendiri?

☐1= Tidak memerlukan bantuan (mencuci lantai dan sebagainya)

☐2= Dengan sedikit bantuan (boleh melakukan kerja-kerja ringan tetapi memerlukan bantuan untuk melakukan kerja-kerja berat)

☐3=Tidak mampu untuk melakukan kerja-kerja rumah

☐4=Saya boleh lakukan tapi saya tidak perlu untuk melaksanakannya (cth. Saya mempunyai orang gaji atau penjaga yang akan melakukan =nya untuk saya)

6. Mengambil ubat?

☐1= Tidak memerlukan bantuan (memakan dos yang betul pada masa yang ditetapkan)

☐2=Dengan sedikit bantuan(boleh mengambil ubat jika ada seseorang menyediakannya untuk anda atau ada seseorang yang mengingatkan anda)

☐3= Tidak mampu untuk mengambil ubat sendiri

☐4= Tidak berkenaan

7. Menguruskan hal-hal kewangan anda?

☐1= Tidak memerlukan bantuan (boleh membayar bil dan menulis cek)

☐2= Dengan sedikit bantuan (menguruskan pembelian harian tetapi memerlukan bantuan seseorang untuk menguruskan pembayaran bil dan menulis cek)

☐3= Atau, adakah anda langsung tidak mampu untuk menguruskan kewangan anda?

8. Melakukan aktiviti berat di sekitar kawasan rumah? (cth: mencuci tingkap dan sebagainya)

☐1= Tidak

☐2= Ya

9. Berjalan naik dan turun dari tangga?

☐1= Tidak

☐2= Ya

10. Berjalan sejauh 1batu (tanpa bantuan)

☐1= Tidak

☐2= Ya

11. Memandu kereta atau lain-lain kenderaan

☐1= Tanpa bantuan (Saya tiada masalah untuk ke mana sahaja yang saya ingin)

☐2= Dengan sedikit bantuan (saya tidak yakin memandu ke tempat yang tidak biasa, saya memerlukan seseorang bersama saya.)

☐3= Buat masa ini, saya tidak dapat memandu

☐4= Tidak berkenaan

H.3 SOAL SELIDIK AKTIVITI FIZIKAL ANTARABANGSA - IPAQ

8. Dalam tempoh 7 hari yang lepas, berapa harikah anda telah melakukan aktiviti fizikal berat, contohnya mengangkat barang berat, mencangkul, senaman aerobic atau berbasikal laju?

☐ 1=Tidak melakukan aktiviti fizikal berat **(Sila ke soalan 3)**

☐____ 2= Sila masukkan jumlah hari yang telah anda habiskan dalam seminggu untuk melakukan aktiviti fizikal berat.

9. Berapakah masa yang biasa anda guna untuk melakukan aktiviti fizikal berat pada salah satu daripada hari berkenaan?

☐ 1= Tidak tahu/Tidak pasti

☐_____2=Sila masukkan jumlah Jam/Minit sehari

10. Dalam tempoh 7 hari yang lepas, berapa harikah anda telah melakukan aktiviti fizikal sederhana, contohnya mengangkat muatan ringan, mengelap lantai, berbasikal pada kelajuan biasa atau bermain badminton beregu? Tidak termasuk berjalan kaki.

☐ 1=Tidak melakukan aktiviti fizikal sederhana (Sila ke soalan 5)

☐_____2=Sila masukkan jumlah hari yang telah anda habiskan dalam seminggu untuk melakukan aktiviti fizikal sederhana.

11. Berapakah masa yang biasa anda guna untuk melakukan aktiviti fizikal sederhana pada salah satu daripada hari berkenaan?

☐ 1=Tidak tahu/Tidak pasti

☐_____2=Sila masukkan jumlah Jam/Minit sehari

12. Dalam tempoh 7 hari yang lepas, berapa harikah anda telah berjalan kaki sekurang-kurangnya 10 minit pada sesuatu masa?

☐ 1=Tidak melakukan aktiviti berjalan kaki(Sila ke soalan 7)

☐_____2=Sila masukkan jumlah hari yang telah anda habiskan dalam seminggu untuk melakukan aktiviti 10 minit berjalan kaki.

13. Berapakah masa yang biasa anda gunakan untuk aktiviti berjalan kaki pada salah satu daripada hari berkenaan?

☐ 1=Tidak tahu/Tidak pasti

☐_____2=Sila masukkan jumlah Jam/Minit sehari

14. Dalam tempoh 7 hari yang lepas, berapakah masa yang anda telah gunakan untuk duduk pada sesuatu hari bekerja?

☐ 1=Tidak tahu/Tidak pasti

☐_____2=Sila masukkan jumlah Jam/Minit sehari

I.2. SOAL SELIDIK JATUH

(Penemubual: SILA BACA- Saya ingin bertanya beberapa soalan samaada anda pernah jatuh)

2. Adakah anda pernah jatuh dalam tempoh 12 bulan yang lalu?
   - ☐ 1=Tidak(**Sila ke soalan 2**)
   - ☐ 2=Ya
   - ☐ 3= Tidak tahu (**Sila ke soalan 2**)

BAHAGIAN J: KUALITI HIDUP

J.1 CASP-19

Berikut adalah pernyataan yang biasa digunakan oleh seseorang untuk menerangkan tentang kehidupan mereka atau perasaan mereka.Kami ingin tahu berapa kerap ianya berlaku sekiranya pernyataan ini ada kaitan dengan diri anda. (Sila tanda satu kotak untuk setiap baris)

		1	2	3	4
		Kerap	Kadang-kadang	Tidak kerap	Tidak Pernah
viii.	Umur saya menghadkan saya daripada melakukan perkara-perkara yang saya inginkan	☐	☐	☐	☐
ix.	Saya rasa apa yang berlaku pada diri saya adalah di luar kawalan saya	☐	☐	☐	☐
x.	Saya berasa bebas untuk merancang masa depan saya	☐	☐	☐	☐
xi.	Saya rasa ketinggalan (terpinggir)	☐	☐	☐	☐
xii.	Saya boleh lakukan perkara-perkara yang saya ingin lakukan	☐	☐	☐	☐
xiii.	Tanggungjawab terhadap keluarga menghalang saya daripada melakukan apa yang ingin saya lakukan	☐	☐	☐	☐
xiv.	Saya rasa puas hati dengan apa yang boleh saya lakukan	☐	☐	☐	☐

		1	2	3	4
		Sering/Selalu	Kadang-kadang	Jarang-jarang	Tidak Pernah
viii.	Kesihatan saya menghalang saya daripada melakukan sesuatu perkara yang ingin saya lakukan	☐	☐	☐	☐
ix.	Kekurangan wang menghalang saya daripada melakukan perkara-perkara yang ingin saya lakukan	☐	☐	☐	☐
x.	Saya bersemangat menghadapi setiap hari yang saya lalui	☐	☐	☐	☐
xi.	Saya berasa hidup saya amat bermakna	☐	☐	☐	☐
xii.	Saya berasa seronok dengan perkara-perkara yang saya lakukan	☐	☐	☐	☐
xiii.	Saya seronok bila berkumpul dengan orang lain	☐	☐	☐	☐
xiv.	Saya berasa bahagia mengimbau kembali kehidupan saya	☐	☐	☐	☐
xv.	Saya rasa amat bertenaga sekarang	☐	☐	☐	☐
xvi.	Saya memilih untuk melakukan perkara yang belum pernah saya buat sebelum ini	☐	☐	☐	☐
xvii.	Saya berpuas hati dengan kehidupan saya sekarang	☐	☐	☐	☐
xviii.	Saya rasakan bahawa kehidupan ini penuh dengan peluang	☐	☐	☐	☐
xix.	Saya rasakan bahawa masa depan saya begitu baik	☐	☐	☐	☐

BAHAGIAN K: ISU KESIHATAN DAN LATAR BELAKANG KESIHATAN

K.1 Kesihatan anda secara umum

1. Bagaimanakah anda menilai tahap kesihatan anda?
   ☐1= Tidak sihat
   ☐2= Sederhana
   ☐3=Baik
   ☐4= Sangat baik
   ☐5= Cemerlang
   ☐99= Tidak tahu

2. Secara umum jika dibandingkan dengan individu yang seusia dengan anda, bagaimanakah anda menilai tahap kesihatan anda?
   ☐1= Tidak sihat
   ☐2= Sederhana
   ☐3= Baik
   ☐4= Sangat baik
   ☐5= Cemerlang
   ☐99= Tidak tahu

3. Adakah keadaan kesihatan anda sekarang menyebabkan masalah kepada perkara-perkara berikut?
   3.2 Pekerjaan (pekerjaan bergaji)) 1= No ☐2= Yes ☐
   3.2 Kerja-kerja rumah 1= No ☐2= Yes ☐
   3.3 Kehidupan sosial 1= No ☐ 2= Yes ☐
   3.4 Hubungan seks 1= No ☐ 2= Yes ☐
   3.5 Minat dan hobi 1= No ☐ 2= Yes ☐
   3.6 Percutian and kegiatan riadah 1= No ☐ 2= Yes ☐
   3.7 Hubungan kekeluargaan 1= No ☐ 2= Yes ☐

4. Adakah berat badan anda menurun lebih daripada10 paun (4.5kg) disebabkan penyakit?
   ☐1= Tidak
   ☐2= Ya
   ☐66= Tidak mahu menjawab
   ☐99= Tidak tahu

K.2 PENGLIHATAN

4. Adakah anda menggunakan cermin mata atau kanta lekap?
   (Penemuduga: Tidak perlu bertanya sekiranya responden memakai cermin mata)
   - ☐1= Tidak(Sila ke soalan2)
   - ☐2= Ya

   1.1 Apakah jenis cermin mata/kanta lekap anda?
   - ☐1= Cermin mata rabun jauh
   - ☐2= Cermin mata untuk membaca
   - ☐3= Bifokal
   - ☐4= Multifokal

5. Adakah penglihatan anda(termasuk kepada pengguna cermin mata/kanta lekap) …
   (Penemuduga:BACAKAN PILIHAN)
   - ☐1= Cemerlang
   - ☐2= Sangat baik
   - ☐3= Baik
   - ☐4= Biasa
   - ☐5= Tidak baik
   - ☐6= Dikategorikan sebagai buta
   - ☐66=Tidak mahu menjawab
   - ☐99= Tidak tahu

6. Berapa baikkah penglihatan anda untuk melihat sesuatu objek yang jauh seperti melihat rakan anda melintas jalan (ini termasuk pengguna cermin mata/kanta lekap). Adakah anda mengatakan penglihatan anda adalah…
   (Penemuduga: BACAKAN PILIHAN)
   - ☐1= Cemerlang
   - ☐2= Sangat baik
   - ☐3= Baik
   - ☐4= Sederhana
   - ☐5= Tidak baik
   - ☐66= Tidak mahu menjawab
   - ☐99= Tidak tahu

7. Adakah doktor pernah memberitahu anda bahawa anda mempunyai masalah penglihatan seperti yang dinyatakan dibawah?
(Penemuduga: BACAKAN PILIHAN)
☐1= Tiada (Tiada masalah penglihatan)
☐2= Glaukoma(tekanan dalam bebola mata)
☐3= Degenerasi makular disebabkanlanjut usia
☐4=Lain-lain
☐5= Katarak**(Sila ke soalan 5)**
☐66= Tidak mahu menjawab
☐88= Tidak tahu

5.Adakah anda pernah menjalani pembedahan membuang katarak?
☐1= Tidak
☐2= Sebelah mata
☐5= Kedua belah mata
☐66= Tidak mahu menjawab
☐88= Tidak tahu

K.3 PENDENGARAN

1. Adakah anda mempunyai sebarang masalah pendengaran?
☐1= Tidak (Sila ke soalan 1.3)
☐2= Ya (Sila ke soalan 1.1)

1.1 Adakah anda menggunakan alat bantuan pendengaran?
☐1= Tidak (Sila ke soalan1.2)
☐2= Ya (Sila ke soalan1.3)

1.2 Jika anda tidak menggunakan alat bantuan pendengaran, sila nyatakan mengapa…
☐1= Tiada sebab
☐2= Sayatidak memerlukannya
☐3= Harganya terlalu mahal
☐4= Alatan tersebut susah untuk digunakan
☐5= Saya tidak suka menggunakannnya
☐6= Lain-lain

1.3 Adakah tahap pendengaran anda … (kepada sesiapa yang menggunakan ataupun tidak menggunakan alat bantuan pendengaran)
(Penemuduga: BACAKAN PILIHAN)
☐1= Cemerlang
☐2= Sangat baik
☐3= Baik
☐4= Sederhana
☐5= Tidak baik
☐66= Tidak mahu menjawab
☐88= Tidak tahu

K.4 DAYA INGATAN

1. Bagaimanakah anda menilai daya ingatan anda dari hari ke hari? Adakah anda merasakan daya ingatan anda adalah….
   (Penemuduga: BACAKAN PILIHAN)
   - ☐1= Cemerlang
   - ☐2= Sangat baik
   - ☐3= Baik
   - ☐4= Sederhana
   - ☐5= Tidak baik
   - ☐66= Tidak mahu menjawab
   - ☐88= Tidak tahu

2. Berapa kerapkah anda merasakan diri andalupa misalnya terlupa dimana anda letakkan cermin mata atau kunci kereta atau terlupa tujuan utama anda masuk ke sesebuah bilik ? Bolehkah anda mengatakan bahawa anda adalah seorang yang pelupa?
   (Penemuduga: BACAKAN PILIHAN)
   - ☐1= Sepanjang masa
   - ☐2= Setiap masa
   - ☐3= Kadang-kadang
   - ☐4= Tidak
   - ☐66= Tidak mahu menjawab
   - ☐88= Tidak tahu

3. Pernahkah anda diberitahu oleh keluarga atau rakan-rakan anda bahawa kekuatan ingatan anda semakin lemah atau mereka memberitahu anda bahawa anda adalah seorang pelupa?
   (Penemuduga: BACAKAN PILIHAN)
   - ☐1= Sepanjang masa
   - ☐2= Kebanykanmasa
   - ☐3= Kadang-kadang
   - ☐4= Tidak
   - ☐66= Tidak mahu menjawab
   - ☐88= Tidak tahu

K.5 LATAR BELAKANG PERUBATAN

Adakah doktor pernah memberi tahu anda bahawa anda menghidapi penyakit-penyakit seperti dibawah? **(Penemuduga: BACAKAN)**

**1.**	**Sakit Jantung**	Tidak<br><br>(1)	Dalam setahun yang lepas<br>(2)	Lebih daripada setahun yang lepas<br>(3)	Tidak Tahu<br><br>(4)
	a. Serangan jantung	☐	☐	☐	☐
	b. Angina	☐	☐	☐	☐
	c. Denyutan jantung tidak tetap	☐	☐	☐	☐
	d. Tekanan darah tinggi	☐	☐	☐	☐
	e.Kolestrol yang tinggi	☐	☐	☐	☐
	f. Jantung terhenti (Jantung berhenti dan memerlukan bantuan pernafasan)	☐	☐	☐	☐
	g. Lemah Jantung	☐	☐	☐	☐
	f. Prosedur jantung (eg. Angioplasti/ endarterektomi / sten)	☐	☐	☐	☐
	g. Pintasan jantung (Pembedahan jantung terbuka/"bypass"	☐	☐	☐	☐
	h. Perentak jantung (alat pacemaker)	☐	☐	☐	☐
	i. Lain-lain masalah yang disebabkan oleh jantung (Sila nyatakan____________________)	☐	☐	☐	☐
**2.**	**Gejala/tanda-tanda penyakit jantung**				
	a. Sakit dibahagiandada >30 minit	☐	☐	☐	☐
	b. Rasa sakit atau tidak selesa dibahagian dada?	☐	☐	☐	☐
	c. Palpitasi (Perasaan jantung berdebar-debar).	☐	☐	☐	☐
**3.**	Sejarah keturunan (keluarga terdekat) penyakit kardiovaskular*ataukematian mengejut disebabkan oleh penyakit kardiovaskular*<br>*Termasuk kardiak, strok(angin ahmar), atau lain-lain penyakit salur darah.	☐idak	☐ Ya		

Adakah doktor pernah memberi tahu anda bahawa anda menghidapi penyakit-penyakit seperti dibawah? **(Penemuduga: BACAKAN)**

**4.**	**Penyakit yang disebabkan oleh sistem pernafasan**	Tidak<br>(1)	Dalam setahun yang lepas<br>(2)	Lebih daripada setahun yang lepas<br>(3)	Tidak Tahu<br>(4)
	a. Asma	☐	☐	☐	☐
	b. Bronkitis	☐	☐	☐	☐
	c. Emfisima/COPD – Penyakit pesaluran pernafasan kronik	☐	☐	☐	☐
**5.**	**Gejala/tanda-tanda penyakit yang disebabkan oleh sistem pernafasan**				
	a. Sesak nafas/semput (apabila berjalan di tanah rata)	☐	☐	☐	☐
	b. Penghasilan kahak pada waktu pagi	☐	☐	☐	☐
	c. Batuk/Penghasilan kahak >3 bulan keatas sehingga 4 tahun	☐	☐	☐	☐

**6.**	**Penyakit Cerebrovaskular**	Tidak<br>(1)	Dalam setahun yang lepas<br>(2)	Lebih daripada setahun yang lepas<br>(3)	Tidak Tahu<br>(4)
	a. Strok(angina ahmar)(rujuk lampiran)	☐	☐	☐	☐
	Jika ianya berlaku sila nyatakan tahun kejadian:		__ __ __ __	__ __ __ __	
	a.1. Jika Ya, berapa kalikah anda mengalami kejadian strok tersebut?		__________<br>(Jumlah kejadian stroke)		
	b. Serangan mini strok/TIA* (Gejala strok yang berlaku selama beberapa minit sehingga 24jam)	☐	☐	☐	☐
	Jika ianya berlaku sila nyatakan tahun kejadian:		__ __ __ __	__ __ __ __	
	b.1. Jika Ya, berapa kalikah anda mengalami kejadian TIA tersebut?		__________<br>(Jumlah kejadian TIA)		

7.	**Lain-lain Penyakit Saraf**	Tidak<br><br>(1)	Dalam setahun yang lepas<br>(2)	Lebih daripada setahun yang lepas<br>(3)	Tidak Tahu<br><br>(4)
	a. Sawan(termasuk epilepsi)	☐	☐	☐	☐
	b. kecederaan otak (disebabkan olehkecederaan di kepala)	☐	☐	☐	☐
	c. Demensia (Nyanyuk)<br>Sila nyatakan jenis (jika tahu):<br>___________________________	☐	☐	☐	☐
	d. Penyakit Parkinson	☐	☐	☐	☐
	e. Lain-lain penyakit (sila nyatakan)<br>_________________________________<br>(cth:kerosakan saraf di anggota badan)	☐	☐	☐	☐

.	**Malignansi**	Tidak<br><br>(1)	Dalam setahun yang lepas<br>(2)	Lebih daripada setahun yang lepas<br>(3)	Tidak Tahu<br><br>(4)
	a. Kanser/barah	☐	☐	☐	☐
	b.Jika YA,				
	i. Payudara	☐	☐	☐	☐
	ii. Paru-paru	☐	☐	☐	☐
	iii.Usus	☐	☐	☐	☐
	iv. Nasofarinks	☐	☐	☐	☐
	v. Lain-lain (sila nyatakan)<br>_________________________________	☐	☐	☐	☐

9.	**Lain-lain masalah kesihatan**	Tidak<br><br>(1)	Dalam setahun yang lepas<br>(2)	Lebih daripada setahun yang lepas<br>(3)	Tidak Tahu<br><br>(4)
	a. Kencing manis	☐	☐	☐	☐
	b.KekuranganVitamin B12	☐	☐	☐	☐
	c. Sakit pada bahagian kaki apabila berjalan akibat salur darah tersumbat)	☐	☐	☐	☐
	d. Penyakit kronik /kegagalan buah pinggang	☐	☐	☐	☐
	e. Penyakit Tiriod (kurang aktif atau hiperaktif)	☐	☐	☐	☐
	f. Ketidakhadaman (Gastroesophageal Reflux/Ulser perut/Ulser duodenal)	☐	☐	☐	☐
	g. Penyakit hati (Hepatitis B/C/D/E/Sirosis)	☐	☐	☐	☐
	h. Artritis (sakit sendi) (Osteoartritis/Reumatoid Arthritis/Lain-lain:______________)	☐	☐	☐	☐
	i. Osteoporosis, kadangkala disebut tulang rapuh	☐	☐	☐	☐
	j. Gout (asid uric tinggi)	☐	☐	☐	☐
	k. Tekanan	☐	☐	☐	☐
	l. Kebimbangan	☐	☐	☐	☐
	m. Lain-lain penyakit psykiatrik (sila nyatakan)____________	☐	☐	☐	☐

n. Inkontinens

Urin:

i. Adakah anda pernah dengan tidak sengaja membuang air kecil ketika anda batuk?	☐	☐	☐	☐
ii. Adakah anda pernah dengan tidak sengaja membuang air kecil sebelum anda sampai di tandas?	☐	☐	☐	☐

o. Lain-lain (sila nyatakan- gunakan lembaran tambahan jika perlu)

______________________________	☐	☐	☐	☐
______________________________	☐	☐	☐	☐

10. Informasi tabiat merokok dan minuman alkohol

a. Adakah anda mengambil minuman beralkohol?

☐ 1= Tidak ☐ 2= Ya ☐ 3= Tidak pernah ☐ 4= Saya pernah

i. Jika ya, berapa kerapkah anda mengambilnya?:

☐ 1= Harian/hampir setiap hari ☐ 2= Hujung minggu sahaja ☐ 3= Sekali-sekala dalam satu bulan ☐ 4= semasa perayaan/majlis sosial

ii.Apakah jenis minuman yang anda selalu ambil?

☐ 1=Bir, 'lager'

☐ 2=Sherry ,wine

☐ 3=Spirits(minuman yang mempunyai peratusan alcohol tinggi)

☐ 4= Pelbagai jenis bir, wine atau spirit

☐ 5=Minuman beralkohol rendah

iii. Satu minuman adalah SEPARUH pain bir, SATU whisky, gin dll, atau satu gelas wine atau sherry. Berapa banyakkah yang anda minum dalam sehari?

☐ 1= Lebih dari 6 minuman sehari

☐ 2=3-6 minuman sehari

☐ 3=2 minuman atau kurang sehari

☐ 4=Tiada

iv. Berapa banyakkah minuman beralkohol yang anda minum seminggu? __________minuman

v. Jika anda pernah minum, Bilakah anda berhenti minum minuman beralkohol? ☐☐

Berapa tahunkah anda telah berhenti minum minuman beralkohol? ☐☐

vi. Mengapakah anda berhenti minum minuman beralkohol?

____

vii. Berapa banyakkah minuman beralkohol yang anda minum dalam seminggu?
__________minuman

b. Sejarah Merokok

Adakah anda merokok? ☐ 1= Tidak ☐ 2=Ya ☐ 3= Tidak pernah ☐ 4= Pernah merokok

i.Jika ya,berapa batang rokok yang anda hisap da sehari ☐☐

ii.Jika anda pernah merokok, pada umur berapakah anda berhenti? ☐☐ Bilangan tahun anda merokok ☐☐

Bilangan rokok yang anda hisap dalam sehari ☐☐

iii. Adakah pasangan anda merokok? ☐ 1= Tidak ☐ 2= Ya ☐ 3=Tidak tahu ☐ 4= Pernah merokok

iii. Jika ya; Berapa kali sehari? ☐☐

Berapa tahunkah pasangan anda merokok? ☐☐

iv. Adakah anda merokok dengan bahan yang lain? ☐ 1=Paip ☐ 2= Cerut ☐ 77= Lain-lain: ____________

11. Sejarah Ubat-ubatan

PENEMUBUAL: BACAKAN: Sekarang saya ingin catatkan semua ubat yang anda pernah ambil secara berkala, seperti setiap hari atau setiap minggu. Ini termasuk ubat preskripsi dan bukan preskripsi, ubat yang dibeli dari farmasi, vitamin, jamudan ubat alternatif.

PENEMUBUAL: Minta responden untuk tunjukkan ubat-ubatan mereka supaya anda dapat menyalin nama ubat-ubatan tersebut dengan ejaan yang betul.

1.	
2.	
3.	
4.	
5.	
6.	
7.	
8.	
9.	
10.	
11.	
12.	
13.	
14.	
15.	

BAHAGIAN L: PENGGUNAAN PENJAGAAN KESIHATAN

L.1 RAWATAN PESAKIT LUAR (UNTUK PENYAKIT AKUT ATAU PEMERIKSAAN KESIHATAN BERKALA).

4. Untuk sebarang penyakit yang anda hidapi dalam tempoh 12 bulan yang lalu, adakah anda pernah pergi ke pusat-pusat kesihatan yang disenaraikan dibawah dan berapa kerapkah lawatan tersebut dibuat?
   (Pilihan pelbagai, Angka)
   ☐1=Tiada(Sila ke soalan 2) ________
   ☐2=Hospital Kerajaan ________
   ☐3=Hospital Swasta ________
   ☐4=Klinik Kesihatan Kerajaan ________
   ☐5=Klinik Swasta ________
   ☐6=Tradisional/pengamal perubatan alternatif ________

5. Apakah sebabnya jika anda tidak pergi untuk mendapat rawatan atau berjumpa dengan doctor dalam tempoh 12 bulan yang lalu?
   (Pilihan pelbagai)
   ☐1=Penyakit tidak cukup serius
   ☐2=Tiada sesiapa yang ingin/boleh membawa saya
   ☐3=Tiada pengangkutan
   ☐4=Terlalu jauh
   ☐5=Kos yang tinggi (cth: kos perubatan, kos pengangkutan)
   ☐6=Takut untuk pergi ke hospital
   ☐7=Tiada siapa yang ingin membantu saya
   ☐8= Merawat/mengubati sendiri penyakit tersebut
   ☐77= Lain-lain (Sila nyatakan _____________)

6. Siapakah yang membayar kos perubatan anda dalam tempoh 12 bulan yang lal?
   (Pilihan pelbagai)
   ☐1=Sendiri
   ☐2=Saudara
   ☐3=Majikan
   ☐4=Badan Kebajikan(bantuan social atau organisasi)
   ☐5=NGO atau Institusi keagamaan
   ☐6=Insurans
   ☐66=Tidak mahu menjawab
   ☐88=Tidak tahu
