# Supplementary material for: Prevalence, risk factors and health outcomes associated with polypharmacy among urban community-dwelling older adults in multi-ethnic Malaysia
Source: PLoS One. 2017 Mar 8;12(3):e0173466. doi: 10.1371/journal.pone.0173466 (PMC5342241; doi:10.1371/journal.pone.0173466)
Supplement: S1 Table — (PDF) [file pone.0173466.s004.pdf]

Supplementary Table 1: Prevalence of medication classes used according to the Anatomical Therapeutic Chemical (ATC) Classification System (ATC first level) among 1256 urban-community dwelling older adults.

ATC first level	Anatomical groups	Number (%)
**C**	Cardiovascular system	1030 (82.0)
**A**	Alimentary tract and metabolism	860 (68.5)
**B**	Blood and blood forming organs	371 (29.5)
**M**	Musculo-skeletal system	276 (22.0)
**N**	Nervous system	164 (13.1)
**Others**	Dietary supplements which were not classified	153 (12.2)
**R**	Respiratory system	95 (7.6)
**G**	Genito urinary system and sex hormones	64 (5.1)
**S**	Sensory organs	41 (3.3)
**H**	Systemic hormonal preparations, excluding sex hormones and insulins	38 (3.0)
**V**	Various	36 (2.9)
**D**	Dermatologicals	22 (1.8)
**L**	Antineoplastic and immunomodulating agents	18 (1.4)
**J**	Antiinfectives for systemic use	16 (1.3)
**P**	Antiparasitic products, insecticides and repellents	1 (0.1)
