# Supplementary material for: Prevalence, risk factors and health outcomes associated with polypharmacy among urban community-dwelling older adults in multi-ethnic Malaysia
Source: PLoS One. 2017 Mar 8;12(3):e0173466. doi: 10.1371/journal.pone.0173466 (PMC5342241; doi:10.1371/journal.pone.0173466)
Supplement: S2 Table — Details of the products taken by the 715 dietary supplements users. (PDF) [file pone.0173466.s005.pdf]

Supplementary table 2: Details of the products taken by the 715 dietary supplement users

Dietary Supplements Products	Total (%)	Polypharmacy, number (%)	Non-polypharmacy, number (%)
**Total**	715 (100.0)	401 (56.1)	314 (43.9)
**Complementary alternative medicine**	35 (4.9)	16 (45.7)	19 (54.3)
**Dietary supplements**			
**Vitamins**	422 (59.0)	287 (68.0)	135 (32.0)
**Minerals**	196 (27.4)	142 (72.4)	54 (27.6)
**Vitamins & minerals**	115 (16.1)	80 (69.6)	35 (30.4)
**Vitamins &/or minerals & others**	39 (5.5)	24 (61.5)	15 (38.5)
**Others**	427 (59.7)	239 (56.0)	188 (44.0)
